# Ferroptosis-Related Gene GCLC Is a Novel Prognostic Molecular and Correlates with Immune Infiltrates in Lung Adenocarcinoma

**DOI:** 10.3390/cells11213371

**Published:** 2022-10-25

**Authors:** Lianxiang Luo, Zhentao Zhang, Yanmin Weng, Jiayan Zeng

**Affiliations:** 1 The Marine Biomedical Research Institute, Guangdong Medical University, Zhanjiang 524023, China; 2 The Marine Biomedical Research Institute of Guangdong Zhanjiang, Zhanjiang 524023, China; 3 The First Clinical College, Guangdong Medical University, Zhanjiang 524023, China

**Keywords:** ferroptosis, lung adenocarcinoma, prognostic signature, overall survival, GCLC

## Abstract

Ferroptosis, a newly discovered iron-dependent type of cell death, has been found to play a crucial role in the depression of tumorigenesis. However, the prognostic value of ferroptosis-related genes (FRGs) in lung adenocarcinoma (LUAD) remains to be further elucidated. Differential expression analysis and univariate Cox regression analysis were utilized in this study to search for FRGs that were associated with the prognosis of LUAD patients. The influences of candidate markers on LUAD cell proliferation, migration, and ferroptosis were evaluated by CCK8, colony formation, and functional experimental assays in association with ferroptosis. To predict the prognosis of LUAD patients, we constructed a predictive signature comprised of six FRGs. We discovered a critical gene (GCLC) after intersecting the prognostic analysis results of all aspects, and its high expression was associated with a bad prognosis in LUAD. Correlation research revealed that GCLC was related to a variety of clinical information from LUAD patients. At the same time, in the experimental verification, we found that GCLC expression was upregulated in LUAD cell lines, and silencing GCLC accelerated ferroptosis and decreased LUAD cell proliferation and invasion. Taken together, this study established a novel ferroptosis-related gene signature and discovered a crucial gene, GCLC, that might be a new prognostic biomarker of LUAD patients, as well as provide a potential therapeutic target for LUAD patients.

## 1. Introduction

LUAD, a type of non-small cell lung cancer (NSCLC), is the most common subtype of lung cancer worldwide [1,2,3]. With advances in diagnosis, chemotherapy, radiotherapy, molecular biology, and precision medicine, the efficacy in LUAD patients improves significantly, but the 5-year overall survival (OS) in LUAD patients remains very low, indicating that the treatment of LUAD still faces huge challenges [4]. As a consequence, discovering new prognostic biomarkers that could be used to provide prognostic predictions and act as new treatment targets for LUAD patients is crucial.

Ferroptosis, a recently found iron-catalyzed type of regulated cell death, is induced by the imbalance of cellular redox homeostasis, leading to a large amount of lipid peroxidation and finally the accumulation of excessive iron-dependent lipid hydroperoxides to a lethal level, which results in cytological changes [5]. Ferroptosis has been associated with the pathophysiological process of many diseases, including cancers, neurological disorders, ischemia–reperfusion damage, renal injury, and blood diseases, according to a recent study [6]. Furthermore, emerging evidence suggests that ferroptosis plays a critical role in tumor suppression and metastasis control, implying that it has significant promise for cancer treatment and prognosis prediction [7,8,9]. Ferroptosis have a regulatory influence on the progression of tumors such as renal cancer, pancreatic cancer, NSCLC, and diffuse large B-cell lymphoma [10], and as a result, ferroptosis induction has emerged as a viable anti-cancer therapy [11].

In recent years, numerous genes have been discovered as modulators or indicators of ferroptosis in addition to ferroptosis-inducing agents [9,11,12,13,14]. Glutathione (GSH) production pathway activation in NSCLC cells has been shown to reduce ferroptosis in recent studies [15,16]. SLC7A11, a critical gene related to ferroptosis, could negatively regulate the process of ferroptosis because of its role in iron concentration regulation. SLC7A11 is overexpressed in LUAD and is strongly linked to tumor growth [15,17,18,19]. It has been reported that the hub gene AKR1C1 is highly expressed with low ferroptosis levels in NSCLC tumors. However, AKR1C1 has been implicated in many pathways involved in the ferroptosis process and connected with diverse cancer infiltrating immune cells, and knockdown suppresses the development of ferroptosis in NSCLC cells [20]. Meanwhile, some studies have shown that redox imbalance and iron absorption and storage dysfunction are important factors in inducing ferroptosis, which is associated with significantly altered expression of GCLC, SLC7A11, and GPX4 [21]. Moreover, ethyl carbamate induced ferroptosis by inhibiting the expression of GCLC and SLC7A11 to inhibit GSH synthesis [22]. Research suggested that γ-glutamyl-peptide synthesis by GCLC provides GSH-independent protection from cystine starvation-induced ferroptosis in cells with NRF2 activation [23]. What is more, it has been discovered that ferroptosis interacts with immune cells to promote tumor progression. One example is that CD8+ T cells stimulate ferroptosis by downregulating SLC7A11 and SLC3A2 and that ferroptosis increases the anticancer efficacy of immunotherapy, demonstrating that ferroptosis may be a mechanism by which the immune system works [24]. Other studies reveal that long non-coding RNA (lncRNA) is increasingly recognized as a key mediator in the regulation of ferroptosis. For example, cytosolic lncRNA P53RRA can promote ferroptosis through the nuclear chelation of P53 [25,26]. As a consequence, looking into the expression profile of the ferroptosis gene and its predictive significance might lead to novel therapeutic thoughts for LUAD.

For this study, we obtain the mRNA expression profiles and clinical data of LUAD patients from The Cancer Genome Atlas (TCGA) and Gene Expression Omnibus (GEO) databases. Then, based on differentially expressed FRGs with significant prognostic value, we built a prognostic signature model that can be used to predict the risk of LUAD patients. The prediction effectiveness of the model is evaluated by a series of analyses. Finally, after risk stratification analysis of patients, we found an important gene, GCLC, and carried out correlation analysis and experimental verification on it to investigate its potential role and activities in the regulation of ferroptosis in LUAD cell lines. Our findings might be valuable for future research on this subject.

## 2. Materials and Methods

### 2.1. Data Collection

A total of 1064 LUAD patients from three independent datasets were included in this study. The RNA-seq data of 594 patients (59 normal samples and 535 LUAD samples) and the corresponding clinical information, such as gender, age, tumor stage, survival status, survival time, and smoking history, were extracted from the TCGA database (https://portal.gdc.cancer.gov/repository, accessed on 8 April 2021) as the training cohort. In the subsequent analysis, 35 patients in the TCGA-LUAD cohort were removed due to their clinical data being incomplete. Thus, the remaining data (*n* = 500) with complete follow-up information were included in our training data set for further analyses. For external validation, the gene expression files and corresponding clinical data from two independent cohorts (GSE68465, *n* = 462 [27]; GSE68571, *n* = 96 [28]) were downloaded from the GEO database (https://www.ncbi.nlm.nih.gov/geo, accessed on 29 April 2021) and combined as a single validation dataset (29 normal and 528 tumors). Batch effects from non-biological technical biases were corrected with the “ComBat” algorithm of the sva package. In terms of data processing, all gene expression profiles were normalized using the scale method provided in the “limma” R package. Simultaneously, the corresponding FRGs were downloaded from FerrDb (http://www.zhounan.org/ferrdb, accessed on 8 April 2021) [29], which is a web-based consortium that provided a comprehensive and up-to-date database for ferroptosis markers, their regulatory molecules, and associated diseases. Overall, based on the data from multiple data sets, 195 FRGs were included in our analysis. The acquisition of the above data fully complied with the access policies of the TCGA and GEO databases. Approval from the local ethics committee was not needed, because our research was based on public databases and strictly followed the publication guidelines.

### 2.2. Identification of Differentially Expressed Genes in the TCGA Datasets

We utilized the “DESeq2” R package to discover the differentially expressed genes (DEGs, with false discovery rate (FDR) < 0.05 and |log2FC| ≥ 1) associated with ferroptosis between 500 tumor tissues and 59 normal tissues in the training cohort based on the FRGs expression profile. The “EnhancedVolcano” R package generated the volcano figure comparing the expression levels of each DEG in normal and malignant tissues. As in previous research, we calculate the ferroptosis potential index (FPI) between the normal and tumor groups using the expression matrix for the purpose of uncovering the functional functions of ferroptosis [30].

### 2.3. Stepwise Construction of Prognosis Signature

First, a univariate Cox analysis of overall survival (OS) was performed to screen FRGs with significant prognostic values of the TGGA training cohort, and genes with a *p*-value < 0.05 were considered statistically significant and incorporated into the subsequent analysis. The Venn diagram indicated the intersection between DEGs and prognostic ferroptosis genes. On the other hand, to examine the connection between the expression of intersected genes and the survival of LUAD patients, the survival analysis of each gene was performed by the “survival” and “survminer” R package (all *p* < 0.05). Next, the LASSO-penalized Cox regression analysis was applied to the intersected genes to minimize the risk of overfitting [31]. Applying the “glmnet” R package, genes with a potentially high correlation with other genes were excluded with the LASSO algorithm with penalty parameter tuning that was conducted through tenfold cross-validation. The LASSO model created a prognostic gene list with coefficients based on the optimal lambda value. Multiple stepwise Cox regression was utilized to establish the prognostic signature. Protein–protein interactions (PPI) analysis was performed on the genes in the signature using the Search Tool for the Retrieval of Interacting Genes (STRING) web tool (http://string-db.org/, accessed on 29 April 2021). A linear combination of the regression coefficient in the regression model and the gene expression levels was used to calculate the prognostic risk score. The gene expression level and related coefficients may be used to calculate each patient’s risk score, as illustrated in the formula below.

(1) $$\begin{array}{c} \mathrm{Risk}\;\mathrm{score}=\mathrm{esum}\;\left(\mathrm{each}\;\mathrm{gene}’s\;\mathrm{expression}\times \mathrm{corresponding}\;\mathrm{coefficient}\right) \end{array}$$

Each LUAD patient’s related risk score was also calculated. Based on their median risk score, patients in the training cohort were divided into low-risk and high-risk groups. Furthermore, Kaplan–Meier analysis was employed to evaluate the recognition efficiency of the signatures by measuring the survival difference between the two groups. Then, based on the R packages “Rtsne” and “rgl,” t-SNE and PCA analytics were, respectively, performed to investigate the distribution of the high- and low-risk groups. Finally, the R package “timeROC” was used to construct receiver operating characteristic (ROC) curves at one, two, and three years and the corresponding time-dependent area under the curves (AUCs) were computed simultaneously, which was used to evaluate the efficiency of the prognostic signature.

### 2.4. External Validation of the Prognostic Signature

For external validation of the predictive capability and applicability of our established signature, we created validation cohorts by two datasets from the GEO database in the previous processing. In the validation cohorts, the same formula and statistical methods were adopted to validate the prognostic capacity of the gene signature. Each patient in the validation cohort had their risk score determined utilizing the same formula as was used in the training cohort. Patients in the validation cohort were also separated into low- and high-risk subgroups based on their risk scores. The Kaplan–Meier survival curve analysis was used to compare the OS times of the two groups, and the ROC curve was utilized to assess the sensitivity and specificity of the gene signature. Similarly, we also performed t-SNE and PCA analytics to explore the distribution of the different groups respectively.

### 2.5. Independence of the Prognostic Signature from Traditional Clinical Characteristics

For the purpose of studying whether the prognostic signature was independent of other traditional clinical characteristics in predicting the OS of patients with LUAD, such as age, gender, and tumor stage, univariate and multivariate Cox regression analyses were performed. *p* < 0.05 was considered statistically significant. The ROC curve was also utilized to determine the sensitivity and specificity of each clinical feature.

### 2.6. Construction and Evaluation of a Predictive Nomogram Integrating FRG Signature and Clinical Data

After the univariate Cox analysis and multivariate Cox analyses, we selected clinical variables with a *p*-value < 0.05 and prognostic signature as the independent prognostic variables, and they were incorporated into the construction of the nomogram. Utilizing the R package “rms”, the nomogram was designed to integrate the risk score of the model as a prognostic factor to evaluate the predictive probability of 1-, 3-, and 5-year OS. Furthermore, calibration curves were developed to evaluate the agreement between actual and nomogram-predicted results. Random opportunities and excellent ability to forecast survival by the nomogram model are represented by the values 0.5 and 1.0, respectively. Net decision curve analyses demonstrate the benefit of predicting LUAD by the nomogram model.

### 2.7. Functional and Pathway Enrichment Analysis

The DEGs between the high-risk and low-risk groups were then screened out by the “limma” package (criteria: FDR < 0.05 and |log2FC| ≥ 1.1). The Gene Ontology (GO) and Kyoto Encyclopedia of Genes and Genomes (KEGG) pathway enrichment analyses were then performed utilizing “clusterProfiler” packages to find the related pathway and functions of these differential genes.

### 2.8. Tumor Microenvironment and Immune Infiltration Analysis

To quantify the proportions of immune cells, we utilized the “gsva” R package to perform a single-sample gene set enrichment analysis (ssGSEA). Moreover, for the sake of comparing the differences in the results obtained by various immune infiltration algorithms, we also downloaded and visualized the immune infiltration results of the TCGA-LUAD cohort from the TIMER2.0 (Tumor Immune Estimation Resource 2.0, http://timer.comp-genomics.org, accessed on 5 July 2021) [32]. An integrated analysis using the Spearman coefficient and Wilcoxon rank-sum was used to determine the association between the immune cell percentage and risk score. Cox, clutter, and Kaplan–Meier analyses were used to filter immune cells with prognostic significance using the percentage and survival data from the immune cells. Yoshihara et al. developed the ESTIMATE method for predicting tumor purity in TME, which includes stromal score, immune score, and estimate score [33]. The stromal, immune and estimate scores of LUAD patients were estimated by the “ESTIMATE” package. The statistically significant criterion was set at a *p*-value of less than 0.05.

### 2.9. Comprehensive Bioinformatics Evaluation of Key Gene GCLC

After single gene survival analysis, we undertook a follow-up investigation on the intersection gene GCLC. The connection between the GCLC expression level and proportions of immune cells, ESTIMATE score, and clinical characteristics were determined using an integrated analysis employing the Kruskal–Wallis test, Spearman coefficient, and Wilcoxon rank-sum. The TISIDB database (http://cis.hku.hk/TISIDB/index.php, accessed on 20 July 2021) is a web-based integrated repository portal that collects large amounts of human cancer data from the TCGA database [34]. The TISIDB database was applied to investigate the relationships between GCLC expression and immunological or molecular subtypes in various cancer types. *p*-values less than 0.05 were deemed statistically significant.

### 2.10. The Human Protein Atlas (HPA)

The Human Protein Atlas (HPA) (http://proteinatlas.org, accessed on 5 January 2022) is a free database of pictures of protein expression in normal and malignant tissues. The immunohistochemistry images of GCLC were searched in the HPA database to verify the bioinformatics analysis results in our study.

### 2.11. Cell Culture and Transfection

Human NSCLC cells(H358) were purchased from the American Type Culture Collection (ATCC). RPMI-1640 medium (Gibco, GrandIsland, NE, USA) containing 10% fetal bovine serum (FBS; Gibco, GrandIsland, NE, USA) and 1% penicillin–streptomycin (Gibco, GrandIsland, NE, USA) was applied to cells culture in a humidified atmosphere with 5% CO_2_ at 37 °C. GCLC was purchased from Sangon Biotech (Shanghai, China) for silencing the expression of GCLC. In this study, the GCLC-siRNA sequence was as follows: 5′-GCUAAUGAGUCUGACCAUU (dTdT)-3′. Cells were cultured in 12-well plates at a density of 5 × 10^4^ cells/well until 60%–70% cell confluence for transfection. The siRNA Transfection Reagent (Polyplus, Illkirch, France) was transfected into H358 cells for 72 h to a final concentration of 5 nM. Successfully transfected cells were used for trials subsequently.

### 2.12. Cell Proliferation

A Cell Counting Kit-8 (CCK8; Beyotime Biotechnology, Shanghai, China) was conducted to examine cell viability according to the manufacturer’s protocols. After being transfected with GCLC-siRNA/ NC-siRNA for 48 h, cells were inoculated in 96-well plates at a density of 4 × 10^3^ cells/well, and we add CCK-8 solution. Then, they were cultured for 0, 24, 48, and 72 h and calculated at 450 nm to determine the number of viable cells.

### 2.13. Colony Formation Assay

The colony formation assay was used to evaluate cell proliferation analysis. Cells were seeded in 12-well plates at a density of 2000 cells/well and incubated at 37 °C and 5% CO_2_ for one week. Later, the cells were rinsed with phosphate-buffered saline (PBS) and fixed in 1 mL/well 4% paraformaldehyde (Leagene Biotechnology, Beijing, China) for 30 min. The dye was then stained with 1% crystal violet solution (Solarbio, Beijing, China) for 20 min at ambient temperature. Eventually, we slowly washed the crystal violet staining solution with PBS and dried it.

### 2.14. Cell Death Assays

Cell death was assessed by flow cytometry following the manufacturer’s instructions. In brief, after transfecting for 72 h, we washed the cells one time with PBS and stained with Annexin V-FITC and PI for 30 min at room temperature in the dark. The measurements are from flow cytometry (BD Biosciences).

### 2.15. Iron Assay

H358 cells were stained with a concentration of 1 μmol/L FerroOrange (Dojindo, Tokyo, Japan) and incubated under 37 °C and 5% CO_2_ conditions for 30 min. Finally, cells were observed under a fluorescence microscope (BioTek Cytation 5, BioTek, Winooski, VT, USA).

### 2.16. Lipid Peroxidation Assay

C11-BODIPY 581/591 (10 µM; ABclonal, Wuhan, China) was added to transfected H358 cells and incubated at 37 °C and 5% CO_2_ for 1 h. After incubating, the cells were washed twice with PBS and digested with trypsin; then, the cells were resuspended in PBS containing 5% FBS and eventually analyzed by flow cytometry. The data were analyzed using FlowJo 10.0.

### 2.17. Glutathione Quantification

The intracellular glutathione (GSH) levels were evaluated using the GSH assay kit (Beyotime, Shanghai, China) according to the manufacturer’s instructions. A total of 5 × 10^5^ H358 cells were seeded in twelve-well plates after treatment with APAP. Harvesting and counting the cells, and the GSH levels were determined using a GSH assay kit.

### 2.18. MDA Assay

The relative concentration of malondialdehyde (MDA) in cells was assessed with a Lipid Peroxidation MDA Assay Kit (Beyotime, Shanghai, China) according to the manufacturer’s instructions. OD values were measured at 532 nm by a microplate reader.

### 2.19. Western Blotting

Proteins were extracted from cells, and cells were lysed with RIPA lysate (Solarbio, Beijing, China) added with PMSF, which was followed by protein quantification using the BCA protein assay kit (Sangon Biotech, Shanghai, China). Proteins were size-fractionated by SDS-PAGE and transferred to nitrocellulose membranes. Membranes were blocked in 5% bovine serum albumin (BSA) for 1 h and then incubated with primary antibodies overnight at 4 °C. The next day, after being washed three times for 15 min in TBST, the membrane was incubated with horseradish peroxidase-labeled secondary antibodies (1:4000) for 1 h at room temperature and then washed three times for 15 min in TBST. Finally, color development was performed using BeyoECL Moon (Beyotime Biotechnology, Shanghai, China).

### 2.20. Statistical Analysis

All data are expressed as mean ± standard deviation (SD). Bioinformatics analyses in this study were performed with R software (v.4.0.3, https://cran.r-project.org/, accessed on 20 January 2022). Statistical analysis was analyzed by using GraphPad Prism analysis software. The *t*-test and Kruskal–Wallis test was used to assess the difference between the two groups and a value of *p* < 0.05 indicates a statistically significant difference, * indicates *p* < 0.05; ** indicates *p* < 0.01; *** indicates *p* < 0.001.

## 3. Results

### 3.1. Identification of Prognostic Ferroptosis-Related DEGs in the Training Cohort

**Figure 1:**
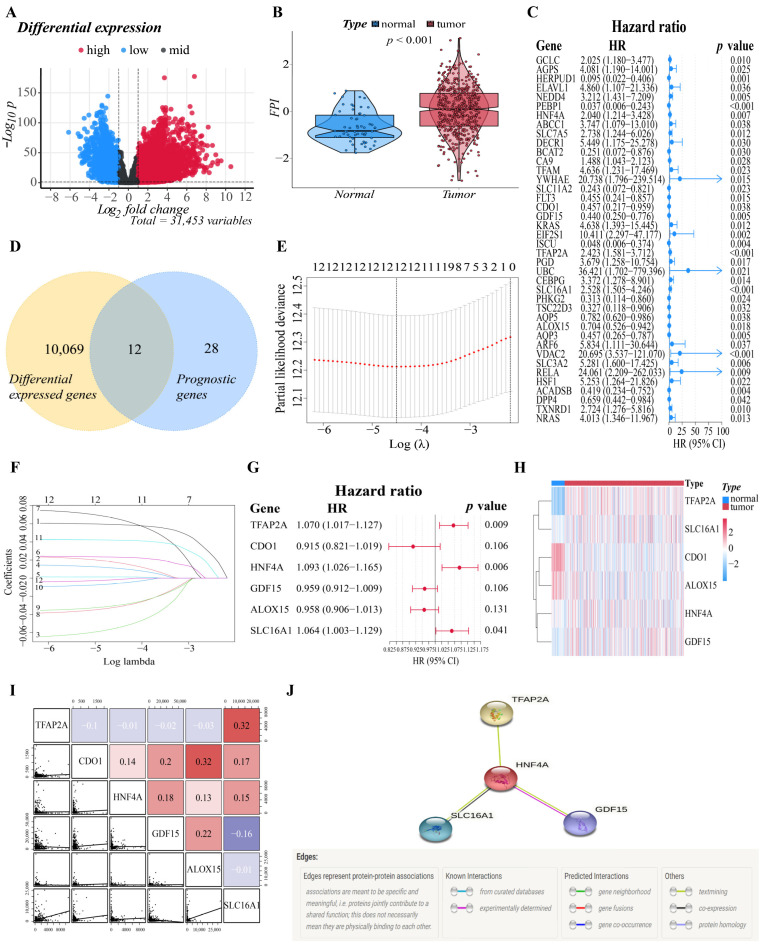
Establishment of the six-gene signature related to ferroptosis. (**A**) The distribution of downregulated and upregulated DEGs is depicted by a volcano map. Blue, black, and red represent the low, equal, and high expression of genes in the relevant group, respectively. The X-axis represents fold change, whereas the Y-axis represents the -log10 FDR value. (**B**) The different FPIs between tumor and normal tissues in LUAD. (**C**) Forest plots showing the results of the univariate Cox regression analysis between gene expression and OS. (**D**) The Venn diagram revealed that 12 genes were intersected between the DEGs set and the prognostic gene set obtained by univariate Cox regression. (**E**) Selecting the optimal λ-value through the cross-validation in the LASSO model. (**F**) LASSO coefficient spectrum of genes enrolled and generate a coefficient distribution map for a logarithmic (λ) sequence. Each curve represents the change trajectory of a single independent variable coefficient. The ordinate represents the coefficient value, the lower abscissa represents log (λ), and the higher abscissa represents the number of non-zero coefficients in the model. (**G**) The forest plot of the multivariate Cox regression analysis. (**H**) A heatmap of the genes in the signature. Blue represents downregulation of genes and red represents upregulation of genes. (**I**) Correlations among TFAP2A, CDO1, HNF4A, GDF15, ALOX15 and SLC16A1 levels in LUAD tissues (TCGA cohort, red: positive correlations; blue: negative correlations). (**J**) The PPI network downloaded from the STRING database indicated the interactions among the candidate genes.

The flowchart of this study is illustrated in Supplementary Figure S1. A total of 500 patients from the TCGA-LUAD cohort and 528 LUAD patients from the GEO cohort were included in the study. To establish a prognostic model that is efficient, we must first identify the genes that are tightly associated with the prognosis of LUAD patients. As a result, we performed difference analysis and univariate Cox analysis first. With an absolute log2-fold change (FC) > 1 and an adjusted *p*-value < 0.05 to perform differential expression analysis, compared with normal tissues (*n* = 59), we identified a total of 10,081 DEGs (7217 upregulated and 2864 downregulated) in TCGA that were related to ferroptosis in LUAD. Nearly one-third of the genes (10,081/31,454, 32.0%) were differentially expressed between tumor samples and normal samples (Figure 1A). On the other hand, a total of 195 FRGs were identified to match the mRNA-sequencing data in the TCGA and GEO databases (Supplementary Table S1). The differences in FPI, a calculated marker of ferroptosis, between tumor and normal tissues were then examined. Significant changes were discovered in the tumor group, with a higher FPI (*p* < 0.001), which illustrated that FPI is closely related to the progression and deterioration of LUAD, and ferroptosis-related prognostic markers may have great potential for the prognosis evaluation of LUAD patients (Figure 1B).

After that, based on 500 LUAD samples with OS rates and survival status in TCGA, we performed univariate Cox regression to investigate the relationship between the expression of the 195 FRGs and prognosis, and the results indicated that 40 genes had significant correlations with the prognosis of LUAD patients (*p* < 0.05). The forest plot showed the results of the univariate Cox regression analysis (Figure 1C). Then, the DEGs obtained from the TCGA datasets were intersected with the prognostic gene set to obtain “differentially expressed prognostic ferroptosis genes”. The Venn diagram revealed that twelve genes were intersected between two gene sets, and they will be included in the subsequent analysis (Figure 1D). The intersected genes were further studied for their prognostic value in LUAD patients. We determined the relationship between the genes’ expression in LUAD and the survival probability of the LUAD patient by survival analysis of the single gene. In both the training and validation cohorts, genes were classified into two groups: high and low expression. After analysis, genes with adjusted P values less than 0.05 were selected for visualization. Supplementary Figure S2 depicted the corresponding Kaplan–Meier survival curves. Analysis showed that the high expression of CA9, GCLC, TFAP2A, SLC12A1, and TXNRD1 correlated with a poor prognosis in LUAD, while the low expression of ALOX15, AQP5, CDO1, and GDF15 correlated with a poor prognosis in LUAD. Significantly, GCLC is included in the results of both cohorts, suggesting that GCLC might be an essential survival-related gene in LUAD.

### 3.2. Construction of the Six-Gene Signatures Related to Ferroptosis

**Figure 2:**
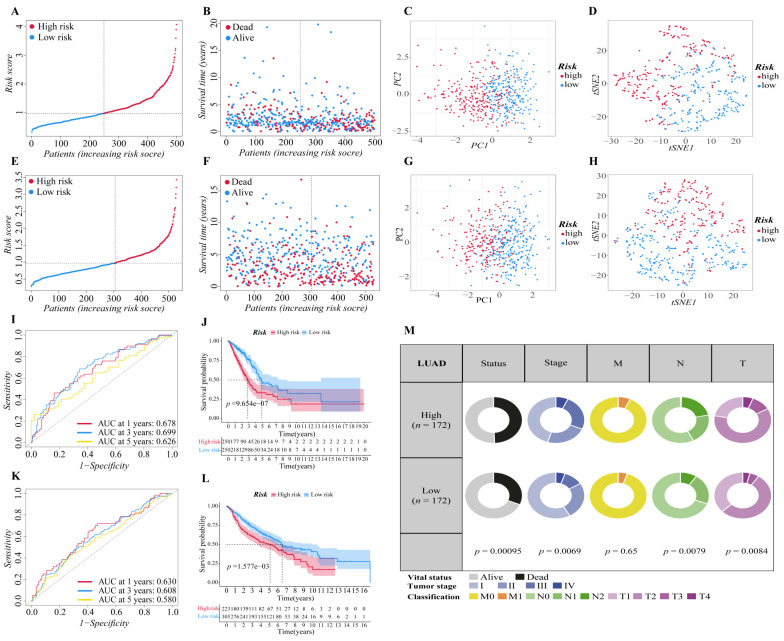
Validation of the six-gene signature in two cohorts. (**A**,**E**) The distribution and median value of the risk scores in two cohorts ((**A**): training cohort; (**E**): validation cohort). (**B**,**F**) The distributions of survival status, overall survival time and risk score ((**B**): training cohort; (**F**): validation cohort). (**C**,**G**) Principal component analysis (PCA) of two cohorts ((**C**): training cohort; (**G**): validation cohort). (**D**,**H**) t-SNE analysis of two cohorts ((**D**): training cohort; (**H**): validation cohort). (**I**,**K**) ROC curve analysis shows the prognostic prediction efficiency of the signature ((**I**): training cohort; (**K**): validation cohort). (**J**,**L**) Kaplan–Meier curves for the OS of patients in the high- and low-risk groups of two cohorts ((**J**): training cohort; (**L**): validation cohort). (**M**) Pie charts showing the Chi-squared test of clinicopathologic factors in LUAD tumor samples from the TCGA.

The above twelve genes were then evaluated again to find the optimum gene combination for developing the prognostic model. We first applied LASSO Cox regression analysis. The best lambda value remains twelve, as calculated by the LASSO regression with 10-fold cross-validation, indicating that these twelve genes have certain independence and no potential significant association (Figure 1E,F). To further identify the FRGs with the greatest prognostic value, we conducted multiple stepwise Cox regressions and determined six FRGs to construct the prognostic signature among LUAD patients (Figure 1G). The heat map shows the difference in the expression of these six genes in the normal and tumor groups of the training cohort, and the results showed that TFAP2A, SLC16A1, HNF4A and GDF15 were upregulated in the tumor group, while CDO1 and ALOX15 were downregulated in the tumor group (Figure 1H). A correlation chart based on the expression patterns of the six genes was also developed to highlight the linkages between them (Figure 1I). In the visualization of the prognostic signatures, to demonstrate the links between the six genes in the prognostic signatures, a PPI analysis was conducted, and the minimum needed interaction score was established at 0.70 (Figure 1J). According to the signatures we established, the risk score of each LUAD patient was measured based on the format: Risk score = (−0.043 * expression level of ALOX15) + (−0.089 * expression level of CDO1) + (−0.041 * expression level of GDF15) + (0.089 * expression level of HNF4A) + (0.062 * expression level of SLC16A1) + (0.068 * expression level of TFAP2A). The patients in two cohorts were then categorized into a high-risk group (training cohort: *n* = 250; validation cohort: *n* = 223) or a low-risk group (training cohort: *n* = 250; validation cohort: *n* = 305) based on the median value of the training cohort’s risk score (Figure 2A,E). After that, we conducted various analyses to assess the effectiveness and stability of the model predictions. As shown in Figure 2B,F, the patients’ death risk increases, and the survival time decreases as the risk score increases, which proves that our prognostic signatures are effective. According to PCA (Figure 2C,G) and t-SNE (Figure 2D,H) analysis, patients in different risk categories were distributed in two separate orientations, which indicated that the risk score we established had a good correlation with the prognosis of LUAD patients. Moreover, the predictive performance of the risk score for OS was evaluated by time-dependent ROC curves, and the area under the curve (AUC) reached 0.678 at 1 year, 0.699 at 3 years, and 0.626 at 5 years (validation cohort: 0.678 at 1 year, 0.699 at 3 years, and 0.626 at 5 years) (Figure 2I,K), indicating better specificity and sensitivity of the prognostic signature in predicting OS. Consistently, the Kaplan–Meier curve demonstrated that patients in the high-risk group had a significantly worse OS than their low-risk counterparts (*p* < 0.05, Figure 2J,L). Clinically, three elements make up the description of the anatomic extent of a lung tumor: T for the extent of the primary tumor, N for lymph node involvement, and M for distant metastases [35]. The TMN classification is a conventional clinical prognostic indicator that can be used to describe tumor progression. Based on the relatively complete clinical data of the training cohort, we compared the survival status, tumor stage, and TMN classification between the high-risk group and low-risk group. The chi-squared test showed that the high-risk group exhibited poorer T classification (*p* = 0.0084), N classification (*p* = 0.0079), stage (*p* = 0.0069), and survival status (*p* < 0.001) than the low-risk group, suggesting that highly malignant LUAD is associated with high predicted values (Figure 2M). According to the results of the preceding investigation, the six-gene signature is trustworthy and valid for prognosis and OS prediction across datasets and platforms.

### 3.3. Independent Prognostic Predictive Efficacy of the Six-Gene Signature

**Figure 3:**
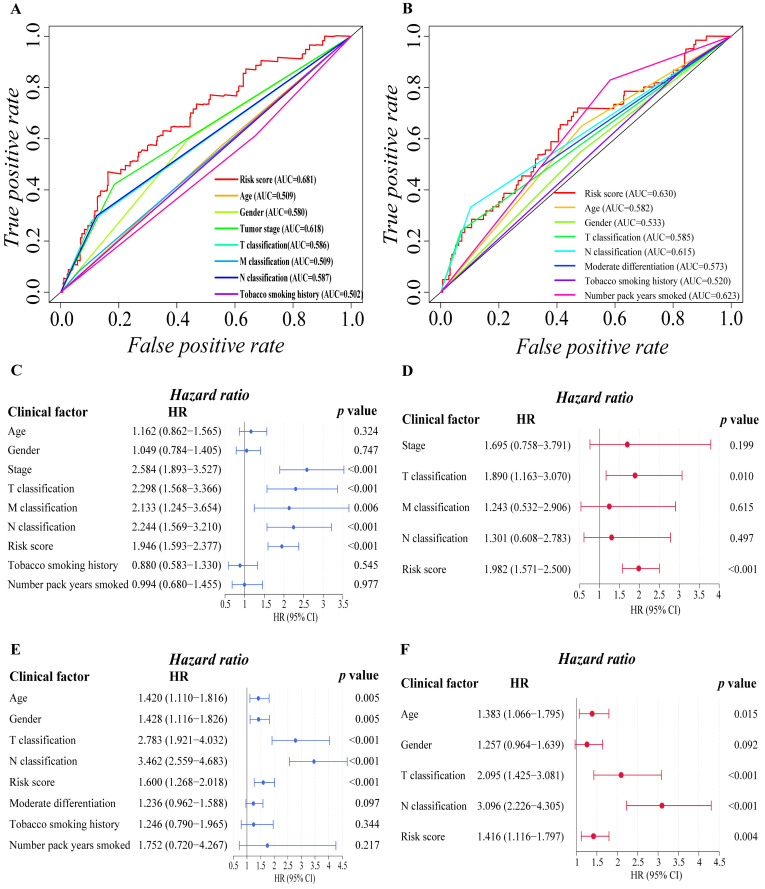
Evaluation of prognostic predictive efficacy of traditional clinical characteristics and six-gene signature. (**A,B**) ROC curve analysis shows the prognostic prediction efficiency of the traditional clinical characteristics ((**A**): training cohort; (**B**): validation cohort). (**C**–**F**) Forest plot of univariate Cox regression analysis ((**C**): training cohort; (**E**): validation cohort) and multivariate Cox regression analysis ((**D**): training cohort; (**F**): validation cohort) of the six-gene prognostic signature and other clinical characteristics.

We proceeded to investigate the independence of the six-gene prognostic signature and traditional clinical characteristics in predicting the prognosis of LUAD patients based on the finding above, ensuring that our model is better than the general clinical characteristics in predicting the prognosis of patients, and it can independently predict the prognosis of patients. Firstly, we preliminarily explored the prognostic value of risk score and various clinical factors through ROC curve analysis (Figure 3A,B). The findings revealed that in addition to the smoking history of the training cohort, additional clinical characteristics of the two cohorts had certain prognostic values. It is worth mentioning that the risk score of the two cohorts showed stronger prognosis predictive power than other clinical variables. Then, univariate and multivariate Cox regression analyses were carried out among the available variables to determine whether the risk score was an independent prognostic predictor for OS. In univariate Cox regression analyses, we found that the tumor stage (HR = 2.584, 95% CI = 1.893–3.527, *p* < 0.001), T classification (HR = 2.298, 95% CI = 1.568–3.366, *p* < 0.001), M classification (HR = 2.133, 95% CI = 1.245–3.654, *p* = 0.006), N classification (HR = 2.244, 95% CI = 1.569–3.210, *p* < 0.001) and risk score (HR = 1.946, 95% CI = 1.593–2.377, *p* < 0.001) in the training cohort were significantly associated with OS (Figure 3C). Similarly, in validation cohort, age (HR = 1.420, 95% CI = 1.110–1.816, *p* = 0.005), gender (HR = 1.428, 95% CI = 1.116–1.826, *p* = 0.005), T classification (HR = 2.783, 95% CI = 1.921–4.032, *p* < 0.001), N classification (HR = 3.462, 95% CI = 2.559–4.683, *p* < 0.001) and risk score (HR = 1.600, 95% CI = 1.268–2.018, *p* < 0.001) were significantly associated with the OS of LUAD patients (Figure 3E). After correction for other confounding factors, the T classification (HR = 1.890, 95% CI = 1.163–3.010, *p* = 0.01) and risk score (HR = 1.982, 95% CI = 1.571–2.500, *p* < 0.001) in the training cohort (Figure 3D), and the age (HR = 1.383, 95% CI = 1.066–1.795, *p* = 0.015), T classification (HR = 2.095, 95% CI = 1.425–3.081, *p* < 0.001), N classification (HR = 3.096 95% CI = 2.226–4.305, *p* < 0.001) and risk score (HR = 1.416, 95% CI = 1.116–1.797, *p* = 0.004) in the validation cohort (Figure 3F) still proved to be independent predictors for OS in the multivariate Cox regression analysis. By and large, the risk score was a reliable independent risk factor connected with OS for LUAD patients in both the training and the validation cohort, and it is more effective than other traditional clinical characteristics in predicting the prognosis of LUAD patients.

### 3.4. Construction and Validation of the Predictive Nomogram

**Figure 4:**
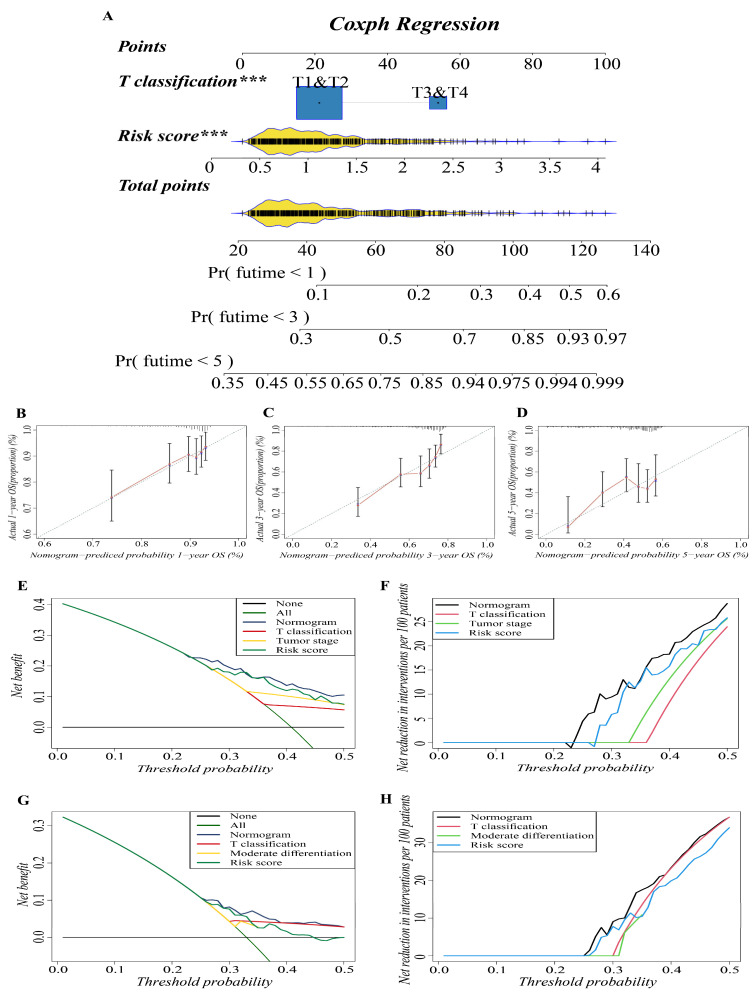
Construction and validation of the predictive nomogram. (**A**) The nomogram for OS prediction at 1, 3 and 5 years was constructed in the training cohort. A value of *p* < 0.05 indicates a statistically significant difference, *** indicates *p* < 0.001. (**B**–**D**) Calibration plots of the nomogram for OS prediction at 1, 3 and 5 years in the training cohort, respectively. (**E**,**G**) Net decision curve analyses demonstrating the benefit of predicting LUAD patients’ prognosis ((**E**): training cohort; (**G**): validation cohort). (**F**,**H**) The net reduction analyses based on nomogram model and other models ((**F**): training cohort; (**H**): validation cohort).

In previous studies, nomograms have been widely used in predicting the prognosis of patients. In this study, we combined our risk-scoring model with clinical traditional clinical features to establish nomograms to further explore the accuracy of prognosis prediction of LUAD patients. T classification and risk score were recognized as important prognostic characteristics in both cohorts based on our study; therefore, we used them to build a predictive nomogram to quantify the prediction results of individual survival probability at 1, 3, and 5 years. As shown in Figure 4A, this nomogram was able to assess two variables to predict a patient outcome, which is based on T classification and risk score. The C index of the OS nomogram was 0.699, and the calibration curves of the nomogram showed great consistency between the predicted OS rates and actual observations at 1, 3, and 5 years; the survival of the nomogram-predicted probability approached the actual survival (Figure 4B–D). In net decision curve analyses for LUAD patients, the nomogram model had a higher net benefit and exhibited a wider range of threshold probabilities in terms of detecting the prognosis of LUAD patients compared with other prediction models (Figure 4E,G). The nomogram model showed a benefit for LUAD threshold probabilities larger than 20%. Net reduction curves, which show the potential to reduce unnecessary intervention (Figure 4F,H), demonstrate that the nomogram model had excellent reduction rates of unnecessary intervention. In general, the nomogram combines the prognosis model we established with the T classification of LUAD patients, and it can be used to predict the prognosis of LUAD patients more accurately.

### 3.5. Functional Enrichment Analyses of Different Risk Groups

**Figure 5:**
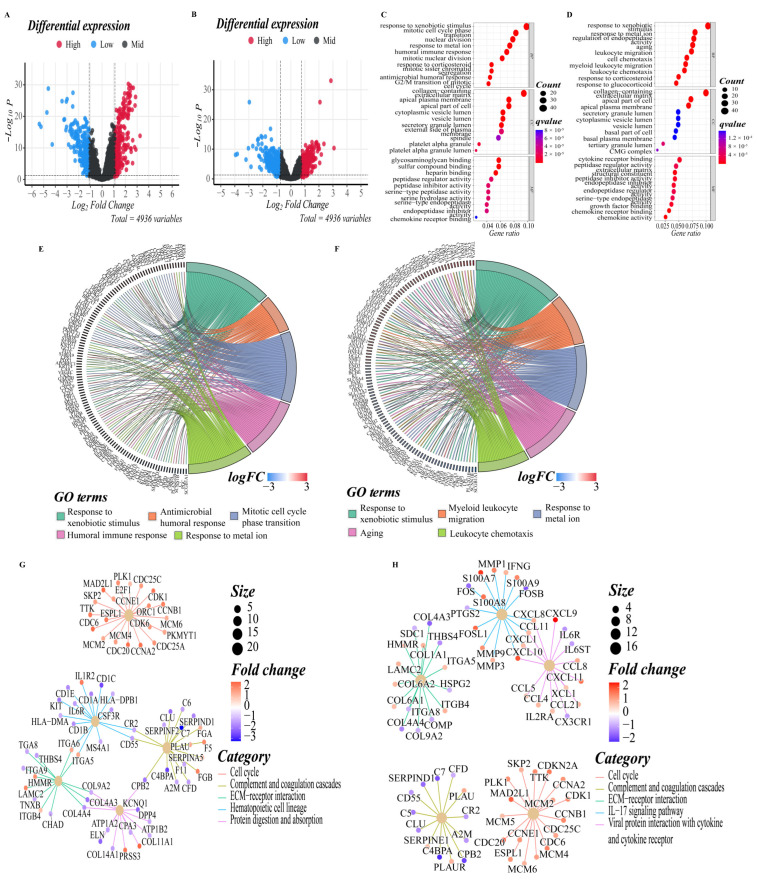
DEGs functional enrichment analysis between high and low-risk groups. (**A**,**B**) The volcano map depicts the distribution of downregulated and upregulated DEGs in the two cohorts’ high-risk and low-risk groups ((**A**): training cohort; (**B**): validation cohort). (**C**,**D**) The point diagram shows the top ten GO terms of each subclass (BP, biological processes; MF, molecular function; CC, cellular component) ((**C**): training cohort; (**D**): validation cohort). (**E**,**F**) The chord plot shows the genes of the top five GO terms of the BP sub-class ((**E**): training cohort; (**F**): validation cohort). (**G**,**H**) The network diagram shows the expression relationship of genes in the top five pathways of KEGG pathway analyses. Fold enrichment of each term is indicated by the x-axis and bar color. ((**G**): training cohort; (**H**): validation cohort).

To elucidate the biological functions and pathways that were associated with the risk score, the DEGs between the high-risk and low-risk groups were used to perform GO enrichment and KEGG pathway analyses (Figure 5A,B). In GO enrichment analyses of the training cohort, DEGs were mainly involved in several biological processes (BP) that are closely related to the tumor progression and immune response, such as response to xenobiotic stimulus, antimicrobial humoral response, mitotic cell cycle phase transition, humoral immune response, mitotic nuclear division, nuclear division, mitotic sister chromatid segregation, and G2/M transition of the mitotic cell cycle, which suggested that these DEGs may be related to the proliferation, invasion and migration of LUAD tumor cells (*p*. adjust < 0.05, Figure 5C,E). In the validation cohort, DEGs were mainly enriched in the activation and migration of leukocytes, such as myeloid leukocyte migration, leukocyte chemotaxis, and leukocyte migration (*p*. adjust < 0.05, Figure 5D,F). It is worth noting that response to metal ion was significantly enriched in both cohorts, implying that there may be an important correlation between high- and low-risk groups and ferroptosis. KEGG pathway analyses on two cohorts indicated that the mainly enriched pathways included the complement and coagulation cascades, cell cycle, ECM–receptor interaction, IL-17 signaling pathway, DNA replication, arrhythmogenic right ventricular cardiomyopathy, viral protein interaction with cytokine and cytokine receptor, PI3K–Akt signaling pathway, etc. The top five enriched pathways for each cohort are shown in Figure 5G,H (*p*. adjust < 0.05). The complete results of KEGG and GO enrichment analyses were shown in Supplementary Tables S2 and S3, which provided references for further research. 

### 3.6. Evaluation of the LUAD Patients’ Immune Microenvironment

**Figure 6:**
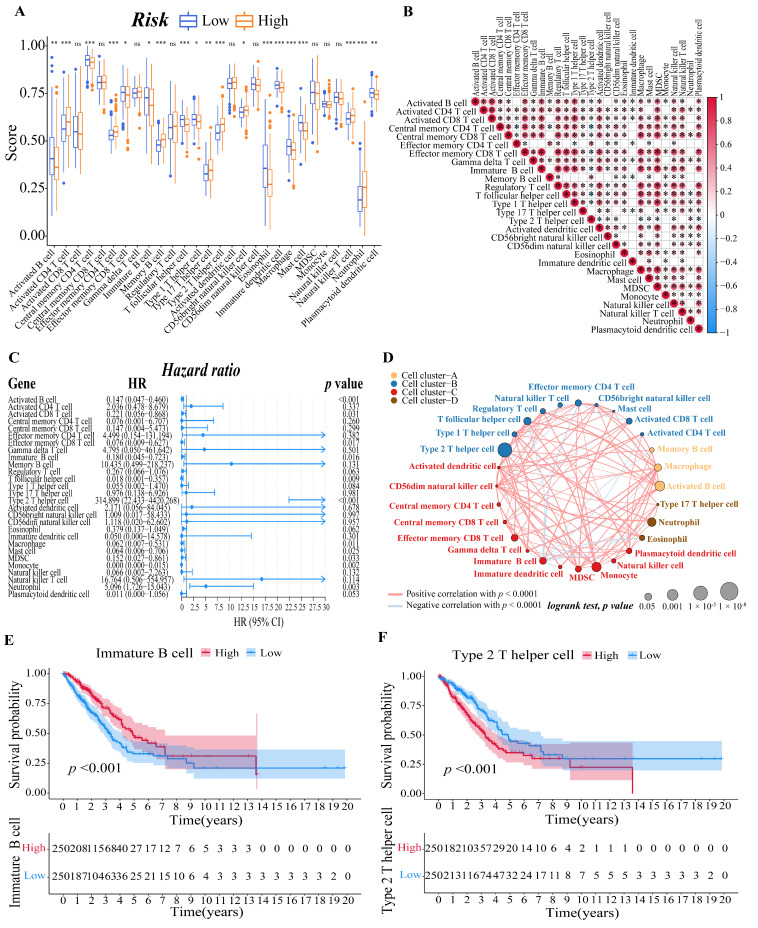
Evaluation of immune microenvironment of LUAD patients. (**A**) Boxplots to display the enrichment scores of 28 immune cells of different risk groups in the training cohort. A value of *p* < 0.05 indicates a statistically significant difference, * indicates *p* < 0.05; ** indicates *p* < 0.01; *** indicates *p* < 0.001. (**B**) Correlation diagram between every two immune cells. The size of the circle and color represents the correlation coefficient R. Red indicates a positive correlation between two immune cells and blue indicates a negative correlation between two immune cells. (**C**) Forest plot of univariate regression Cox analysis based on 28 kinds of immune cells. (**D**) Cellular interaction of tumor microenvironment cell types. The size of each cell represents the survival impact of each immune cell type, which was calculated using the formula log10 (log-rank test *p* value). Cellular interactions are shown by the lines linking immune cells. The strength of connection calculated using Spearman correlation analysis is shown by the thickness of the line. The positive correlation is shown in red, while the negative connection is shown in blue. (**E**,**F**) The corresponding Kaplan–Meier survival curves of the immune cells that are significantly associated with survival ((**E**), immature B cell; (**F**), type 2 T helper cell).

To further explore the correlation between the risk score and immune cells, we quantified the enrichment scores of diverse immune cell subpopulations with the ssGSEA R package. The results showed that the activated B cell, central memory CD4 T cell, effector memory CD8 T cell, immature B cell, T follicular helper cell, type 1 T helper cell, eosinophil, immature dendritic cell, macrophage, mast cell, and plasmacytoid dendritic cell were significantly different between the low-risk and high-risk group, and the scores of them were all lower in the high-risk group. The scores of activated CD4 T cell, effector memory CD4 T cell, memory B cell, type 17 T helper cell, type 2 T helper cell, CD56 bright natural killer cell, natural killer T cell, and neutrophil, on the other hand, were significantly higher in the high-risk group, implying that these immune cells were more active in the high-risk group (*p* < 0.05, Figure 6A). Meanwhile, we visualized the immune infiltration results of the TCGA–LUAD cohort from the TIMER2.0 database to examine the discrepancies in the results generated by various immune infiltration algorithms (Supplementary Figure S3).

Then, we calculated the Pearson correlation coefficient between each immune cell based on the ssGSEA results to estimate the correlation between them (Figure 6B). Moreover, after a univariate Cox analysis of each immune cell (Figure 6C), we draw an immune cell network, which depicted a comprehensive landscape of tumor–immune cell interactions, cell lineages, and their effects on the overall survival of patients with LUAD (Figure 6D). Finally, we determined the relationship between the immune cell and the survival probability of the LUAD patient by survival analysis. We set the threshold of *p*-value as *p* < 0.001, and the immature B cell (Figure 6E) and type 2 T helper cell (Figure 6F) revealed by the data showed they are both significantly associated with survival in LUAD patients.

### 3.7. GCLC Could Be a Significant Prognostic Predictor

**Figure 7:**
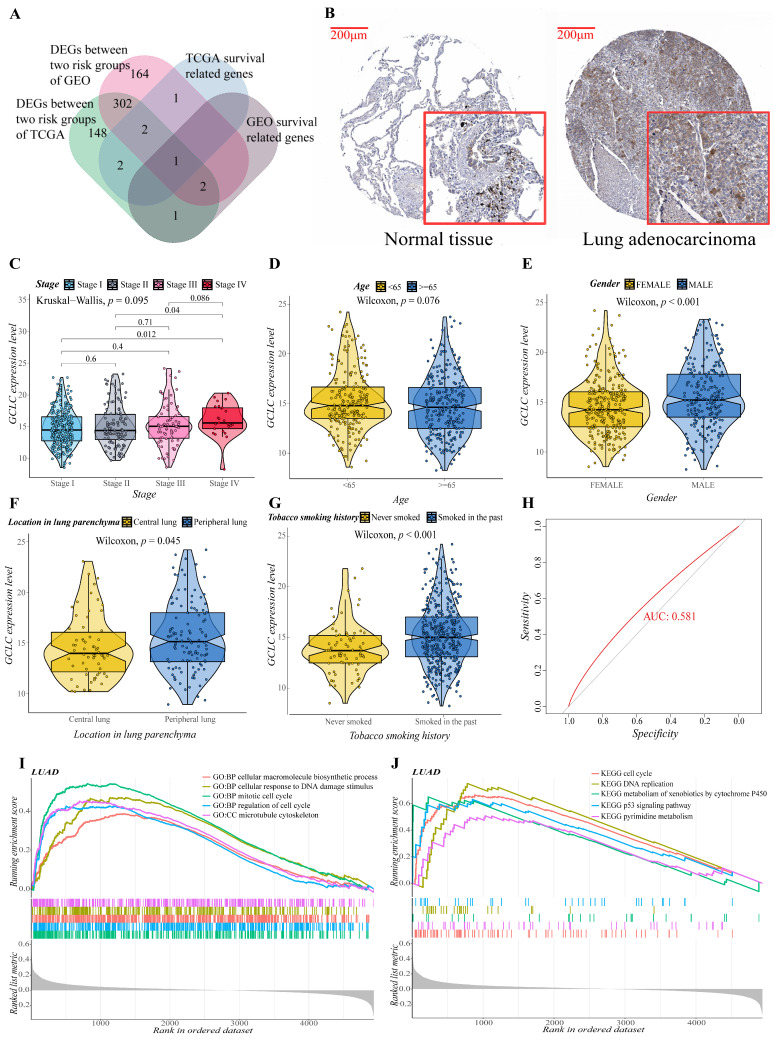
Evaluation of the prognostic value and predictive potential of GCLC. (**A**) Venn diagram of four groups of genes (green: DEGs between two risk groups of training cohort; red: DEGs between two risk groups of validation cohort; blue: survival-related genes of training cohort; brown: survival-related genes of validation cohort). (**B**) GCLC protein expression in LUAD tissue samples and corresponding non-cancer tissue samples. GCLC protein levels were upregulated in LUAD tissues compared to the corresponding non-cancer tissues in the IHC results from the HPA database. (**C**–**G**) Correlation between GCLC expression level and various clinical characteristics ((**C**), tumor stage; (**D**), age; (**E**), gender; (**F**), the location of the tumor in the lung parenchyma; (**G**), tobacco smoking history). The test for association between paired samples used Pearson’s correlation coefficient. Two-tailed statistical *p* values were calculated by a two-sample Mann–Whitney test or Student’s *t*-test when appropriate. (**H**) ROC curve analysis shows the prognostic prediction efficiency of GCLC. (**I**,**J**) GSEA analysis showed the top five GO (**I**) and KEGG (**J**) enrichment results of the high- and low-expression groups of GCLC.

We obtained the intersection gene GCLC of the two cohorts in the single gene survival analysis, suggesting that GCLC may be a significant survival-related gene. It is interesting to note that when the Venn diagram is made (Figure 7A), it is discovered that GCLC is also the differential gene between the two cohorts’ risk groups. As a result, we performed a series of single-gene bioinformatics analyses on GCLC to assess its predictive potential in depth. Firstly, GCLC was discovered as a poor prognostic factor in the aforementioned survival study, and it is highly expressed in LUAD tissue. Therefore, we verified this conclusion by obtaining the IHC images of normal and LUAD tissues from the HPA database. In normal tissue, GCLC was mostly found in macrophages, but alveolar cells were not stained, which was consistent with earlier findings. GCLC also exhibited medium staining in tumor tissue (Figure 7B). Then, we evaluated the correlation between the expression level of GCLC and various clinical characteristics. After analysis, the expression level of GCLC was related to tumor stage (stage I to stage IV and stage II to stage IV, *p* < 0.05, Figure 7C), patients’ gender (*p* < 0.001, Figure 7E), the location of the tumor in the lung parenchyma (*p* < 0.05, Figure 7F) and tobacco smoking history (*p* < 0.001, Figure 7G), but not to the patient’s age (*p* > 0.05, Figure 7D). In summary, in male LUAD patients (classification of gender), LUAD patients with tumors located in the peripheral lung (classification of the location of the tumor in the lung parenchyma), and LUAD patients who smoked in the past (classification of tobacco smoking history), the expression level of GCLC is higher than the LUAD patients in other groups under this classification (*p* < 0.05), which suggested that the expression of GCLC has an important relationship with the occurrence and development of LUAD. Furthermore, ROC curves were used to assess GCLC’s predictive ability for patients’ OS, and the area under the curve (AUC) reached 0.581 (Figure 7H), indicating that GCLC has some utility in predicting patients’ OS. The patients were then divided into two groups based on the median value of GCLC expression, and we used GO and KEGG enrichment analysis to look for the significant enrichment of biological processes and pathways of DEGs between the two groups. As shown in Figure 7I,J, biological processes such as cell cycle process, cellular macromolecule biosynthetic process, cytoskeleton organization, mitotic cell cycle and regulation of cell cycle, pathways such as metabolism of xenobiotics by cytochrome p450, oocyte meiosis, spliceosome, steroid hormone biosynthesis, and ubiquitin-mediated proteolysis were significantly enriched in the GCLC high-expression group, which further proved that the high expression of GCLC promotes tumor growth and metastasis.

**Figure 8:**
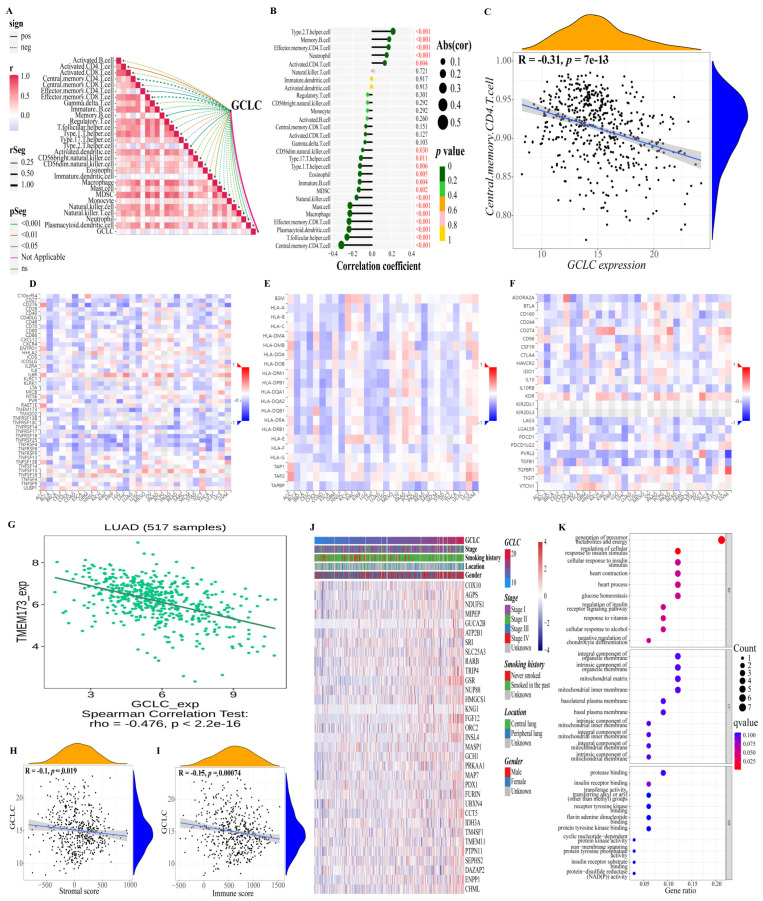
Further study of GCLC in immunity and gene correlation. (**A**,**B**) Correlations between GCLC and 28 kinds of immune cells ((**A**): heat map; (**B**): lollipop graph). (**C**) There is a decent correlation coefficient between central memory CD4 T cell and the expression level of GCLC. (**D**–**F**) The heat map shows the relations between three kinds of immunomodulators ((**D**), Immunostimulator; (**E**), MHC molecule; (**F**), Immunoinhibitor) and the expression of GCLC in Pan-cancer. (**G**) TMEM173 has a strong correlation with the expression of GCLC in patients with LUAD. (**H**,**I**) The correlation between the level of GCLC expression and the tumor stromal cell scores (**H**) and immune cell scores (**I**). (**J**) The heat map shows the relation between the expression of the 33 genes and some LUAD patient clinical characteristics. (**K**) The dot map shows the GO enrichment analysis results of 33 genes.

Moreover, we also studied the relationship between GCLC and the immune microenvironment. Based on the previously obtained immune cell proportion matrix, we calculated the Pearson correlation coefficient between the proportion of each immune cell and the expression level of GCLC (Figure 8A,B). According to the findings, GCLC expression exhibited the highest positive correlation with type 2 T helper cell and the highest negative correlation with central memory CD4 T cell expression. (Figure 8C, *p* < 0.001). Interestingly, the type 2 T helper cell had also been proven to have an important relationship with the survival of LUAD patients in the previous results, suggesting that the type 2 T helper cell may be important cells for LUAD tumor progression. Then, we continue to study the relations between three kinds of immunomodulators (immunostimulator (Figure 8D), MHC molecule (Figure 8E), and immunoinhibitor (Figure 8F)) and the expression of GCLC. The results showed that in LUAD, compared with other immunomodulators, the expression of immunostimulator TMEM173 had a stronger negative correlation with the expression level of GCLC (R = −0.476, *p* < 0.001, Figure 8G). The ER protein TMEM173 was previously identified to enhance ferroptosis in human pancreatic cancer cell lines by enhancing MFN1/2-dependent mitochondrial fusion [36]. Therefore, TMEM173 may be an important immunomodulator to promote ferroptosis in LUAD patients. In addition, we estimated the immune cell and stromal cell scores of each LUAD patient in the training cohort to investigate the impact of GCLC expression on the immune microenvironment. The results revealed that GCLC expression was negatively correlated with stromal cell scores (R = −0.1, *p* < 0.05, Figure 8H) and immune cell scores (R = −0.15, *p* < 0.001, Figure 8I). Moreover, to study the genes highly associated with GCLC, we set the threshold as the correlation coefficient R > 0.5, *p*-value < 0.05 to screen the genes based on the patient’s gene expression profile. The findings revealed that 33 genes were highly correlated to GCLC expression levels. The heat map depicts the relation between the expression of 33 genes and some LUAD patient clinical characteristics (Figure 8J). Furthermore, we performed GO enrichment analysis on these 33 genes to investigate the biological processes, molecular function, and cellular components of gene enrichment. The findings reveal that these highly correlated genes are mainly enriched into two biological processes: regulation of cellular response to insulin stimulus and generation of precursor metabolites and energy (q-value < 0.05, Figure 8K).

### 3.8. GCLC Silencing Promotes Ferroptosis of H358 Cells

**Figure 9:**
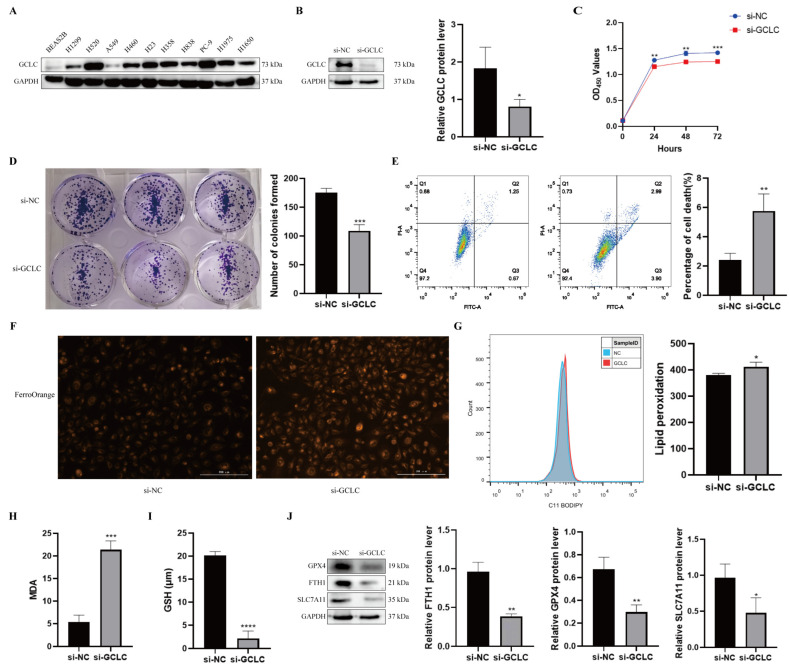
GCLC silencing promotes ferroptosis of H358 cells. (**A**) The expression level of GCLC in BEAS2B cells and NSCLC cell lines by Western blot. (**B**) After H358 cells were transfected with si-GCLC or si-NC for 72 h, the protein expression of GCLC was detected by Western blot assay. * si-GCLC vs. si-NC, * *p* < 0.05, ** *p* < 0.01, *** *p* < 0.001, ****p<0.0001. (**C**,**D**) CCK8 assay and colony formation assay were measured in siNC- or siGCLC-transfected cells. * si-GCLC vs. si-NC, * *p* < 0.05, ** *p* < 0.01, *** *p* < 0.001. (**E**) Cell death assay was determined by flow cytometry in H358 cells. * si-GCLC vs. si-NC, * *p* < 0.05, ** *p* < 0.01, *** *p* < 0.001. (**F**–**I**) Fe^2+^, lipid peroxidation, MDA, and GSH levels were examined in H358 cells transfected with si-NC or si-GCLC. * si-GCLC vs. si-NC, * *p* < 0.05, ** *p* < 0.01, *** *p* < 0.001. (**J**) The expression levels of GPX4, FTH1, and SLC7A11 in H358 cells with GCLC silencing were determined by Western blot. * si-GCLC vs. si-NC, * *p* < 0.05, ** *p* < 0.01, *** *p* < 0.001.

To further evaluate GCLC expression in NSCLC, we examined protein levels of GCLC in human bronchial epithelial cells and NSCLC cell lines. It was found that the protein level of GCLC was much higher in NSCLC lines (Figure 9A). As a consequence, H358 cells were selected for the subsequent experiments. To verify the above conclusion, we examined the biological function of GCLC in H358 cells. GCLC protein expression was significantly downregulated in H358 cells after transfection with siRNA, indicating that GCLC was successfully knocked down (Figure 9B). Then, we performed the CCK8 and the colony formation assay in H358 cells to explore the effect of GCLC on the proliferation of the cells. The results showed that compared to the si-NC group, the proliferation in the si-GCLC group was significantly reduced (Figure 9C,D). In addition, we performed the cell death assay to show whether GCLC affects cell survival. As we expected, the percentage of cell death was significantly increased in the si-GCLC group compared with the si-NC group (Figure 9E).

Furthermore, we studied key indicators of ferroptosis by interfering with GCLC expression in H358 cells to learn more about the role of GCLC expression in ferroptosis. First of all, we discovered that after knocking down GCLC, the level of intracellular Fe^2+^ increased in H358 cells (Figure 9F). Ferroptosis is characterized by lipid peroxidation, which produces malondialdehyde as a byproduct (MDA). As a result, we examined the influence of GCLC on intracellular lipid peroxidation and MDA in H358 cells, discovering that inhibiting GCLC expression enhanced intracellular lipid peroxidation and MDA levels in H358 cells (Figure 9G,H). GSH is an antioxidant that plays a crucial role in maintaining the redox balance and defending against oxidative stress in cells. GSH levels in H358 cells were significantly decreased following GCLC knockdown in comparison with the control group (Figure 9I). What is more, Western blot results showed that GPX4, FTH1, and SLC7A11 levels were decreased in H358 cells after silencing GCLC (Figure 9J). In conclusion, these results suggested that silencing GCLC can cause H358 cells to go into ferroptosis.

## 4. Discussion

Ferroptosis is a novel form of programmed cell death characterized by the excessive accumulation of intracellular iron and an increase in reactive oxygen species (ROS) [5]. This distinct pattern of cell death has been the subject of several research in recent years, and it is widely regarded as a viable treatment option for a variety of cancers [11]. However, research on the precise involvement of ferroptosis in LUAD, as well as its possible mechanisms and routes, is currently rare. In our study, we gradually constructed a prognostic signature consisting of six FRGs and separated LUAD patients into high- and low-risk groups. In the evaluation, the Kaplan–Meier curves and the area under the curve of the ROC curves manifested that the ferroptosis-related signature had fine predictive accuracy, and its good prediction performance is also maintained in the validation cohort. More importantly, in the single gene survival analysis and the risk difference analysis of the model, we found a key gene, GCLC, and its high expression was related to a poor prognosis in LUAD. Moreover, we experimentally verified that silencing GCLC promoted the development of ferroptosis and inhibited the proliferation and invasion of LUAD cells.

In the model we established, a total of six genes (ALOX15, CDO1, GDF15, HNF4A, SLC16A1, TFAP2A) were implicated. Among them, ALOX15 and CDO1 positively regulated ferroptosis, while GDF15, SLC16A1, TFAP2A, and HNF4A negatively regulated ferroptosis. In addition, we evaluated the effectiveness of the model prediction through a variety of bioinformatics analyses and external validation to determine the value of it in clinical application In time-dependent ROC curves analysis, the area under the curve (AUC) is greater than 0.626, indicating the better specificity and sensitivity of the prognostic signature in predicting OS. Moreover, the Kaplan–Meier curve demonstrated that patients in the high-risk group had a significantly worse OS than their low-risk counterparts, which further proves the effectiveness of the model in predicting and classifying the prognosis of LUAD patients. However, the prognostic score model was built based on the TCGA–LUAD cohort, and it is clear that internal validation alone is insufficient and the quality of external verification results is crucial for accurately assessing the model’s ability to make predictions. Compared with the model established in previous LUAD studies [37,38], our model shows better predictive effectiveness in external validation, which suggested that our model has reliable prediction performance for prognosis. In addition, in some subsequent analyses, our model is superior to the traditional clinical features in predicting the prognosis of patients, and significant differences between high-risk and low-risk groups were also found, indicating the good classification effect of the model. Ultimately, our analysis confirmed the model’s accuracy and stability in forecasting the prognosis of LUAD patients.

More importantly, we intersected the differential genes of patients in high-risk and low-risk groups with survival-related genes and obtained an important gene GCLC. The ligation of cysteine with glutamate is catalyzed by GCLC, which is the initial step in glutathione production. Cysteine levels, on the other hand, are tightly regulated due to their reactive thiol moiety and crucial role in redox homeostasis. Although cysteine is directly related to GSH synthesis, cysteine availability can affect the levels of cofactors and metabolites associated with ferroptosis, including the production of coenzyme A [39,40] and iron–sulfur clusters [41]. Several studies have shown that cysteine starvation can impair growth in numerous in vivo cancer models [42,43], and the pharmacological targeting of cystine uptake can effectively cause cancer cell death, such as ferroptosis [5,42,43]. Interestingly, previous studies have found that non-small cell lung cancer (NSCLC) cells are sensitive to cystine starvation [44]. GCLC generates γ-glutamyl-peptides by substituting small, non-charged amino acids for cysteine in the ligation with glutamate when cysteine was deficient. GCLC was also shown to produce γ-glutamyl-peptide in mouse tissues. This promiscuous activity reduced glutamate buildup to defend against ferroptosis [23]. Moreover, in previous studies, GCLC has been reported to be highly expressed in various types of cancer, such as breast cancer [45], hepatocellular carcinoma [46], and colon cancer [47]. High GCLC expression in breast cancer enhances GSH biosynthesis with a concurrent reduction in intracellular ROS accumulation, thereby provoking reductive stress [48]. Likewise, the results of another study also showed that tumor GCLC is a potential prognostic biomarker for HCC patients after receiving curative resection [46]. However, the biological role of GCLC in LUAD has not been clarified.

It is important to mention that we discovered that the type 2 T helper cell was strongly associated with LUAD patients’ survival. The content of the type 2 T helper cell was inversely correlated with patients’ quality of life for LUAD. Additionally, there was a strong correlation between the expression of GCLC and the quantity of type 2 T helper cell in the LUAD tumor tissue. In previous studies, allergen-specific T helper 2 (Th2) cells play central roles in developing allergic asthma [49], but in tumor research, the study of lung cancer immunity has focused almost exclusively on Th1/Th2 cell balance. In the late stage of NSCLC precursor lesion, the content of TH1 cytokines decreases and the content of TH2 and Treg cytokines increases, and these changes are related to tumor immune escape [50]. Therefore, we infer that in the development of LUAD, the upregulation of GCLC expression may lead to the increase in type 2 T helper cell content, thereby promoting tumor immune escape.

In the present study, we found that the high expression of GCLC, a ferroptosis-related gene, was related to a poor prognosis in LUAD. In addition, the results of our experiments show that silencing GCLC inhibited the release of glutathione, increased ferrous ions, malondialdehyde, and lipid peroxidation levels of LUAD cells, promoting the development of ferroptosis and inhibiting the proliferation and invasion of LUAD cells. However, the more complex relationships and regulatory mechanisms among GCLC, ferroptosis, and LUAD still has to be clarified.

Inevitably, there are several limitations in our study. First, because there have been few studies on the role of ferroptosis in malignancies, the information provided by the FerrDb website regarding FRGs may be unreliable, and certain critical ferroptosis-mediating genes may be absent from the ferroptosis gene sets. Second, we built a survival model based on FRGs for making prognostic predictions of LUAD patients using retrospective data from the TCGA database. The model was validated using data from the GEO database that was collected retrospectively. As a result, more prospective data are required to confirm the therapeutic utility of our FRG-based survival model. Furthermore, in the subsequent experiments, we will construct shRNA to stably knock down GCLC and then observe its effect on the tumor for the reason that the si-RNA we used in this experiment can only briefly knock down GCLC. In addition, we will construct xenograft models with nude mice and collect clinical samples for further validation and intensive study.

In conclusion, our study developed a novel prognostic model based on six FRGs, which was found to be independently associated with OS, indicating that it can apply to predict LUAD patients’ OS. In addition, we discovered a key gene, GCLC, which is related to LUAD patients’ prognosis and ferroptosis. Our study may provide insight into the identification of therapeutic targets for LUAD.

## Data Availability

Research data and other items supporting the results in this article are available upon reasonable request to the corresponding author.

## Supplementary Materials

The following supporting information can be downloaded at: https://www.mdpi.com/article/10.3390/cells11213371/s1. Figure S1. Flow chart of data collection and analysis; Figure S2. The corresponding Kaplan–Meier survival curves of the intersected genes. (**A**–**F**) The results of genes with adjusted *p*-value less than 0.05 in the training cohort. (**G**–**J**) The results of genes with adjusted *p*-value less than 0.05 in the validation cohort. The gene that was found in both cohorts’ results is in the red box; Figure S3. The heatmap for immune responses based on TIMER, CIBERSORT, CIBERSORT-ABS, QUANTISEQ, MCPCOUNTER, XCELL, EPIC and ssGSEA algorithms among high and low risk group. Table S1. A total of 195 FRGs were included in our study and entered into the next procedure; Table S2. GO enrichment results of DEGs between the high-risk group and low-risk group of two cohorts; Table S3. KEGG enrichment results of DEGs between the high-risk group and the low-risk group of two cohorts.
