# Supplementary figures and images for: Ferroptosis-Related Gene GCLC Is a Novel Prognostic Molecular and Correlates with Immune Infiltrates in Lung Adenocarcinoma
Source: Cells. 2022 Oct 25;11(21):3371. doi: 10.3390/cells11213371 (PMC9657570; doi:10.3390/cells11213371)

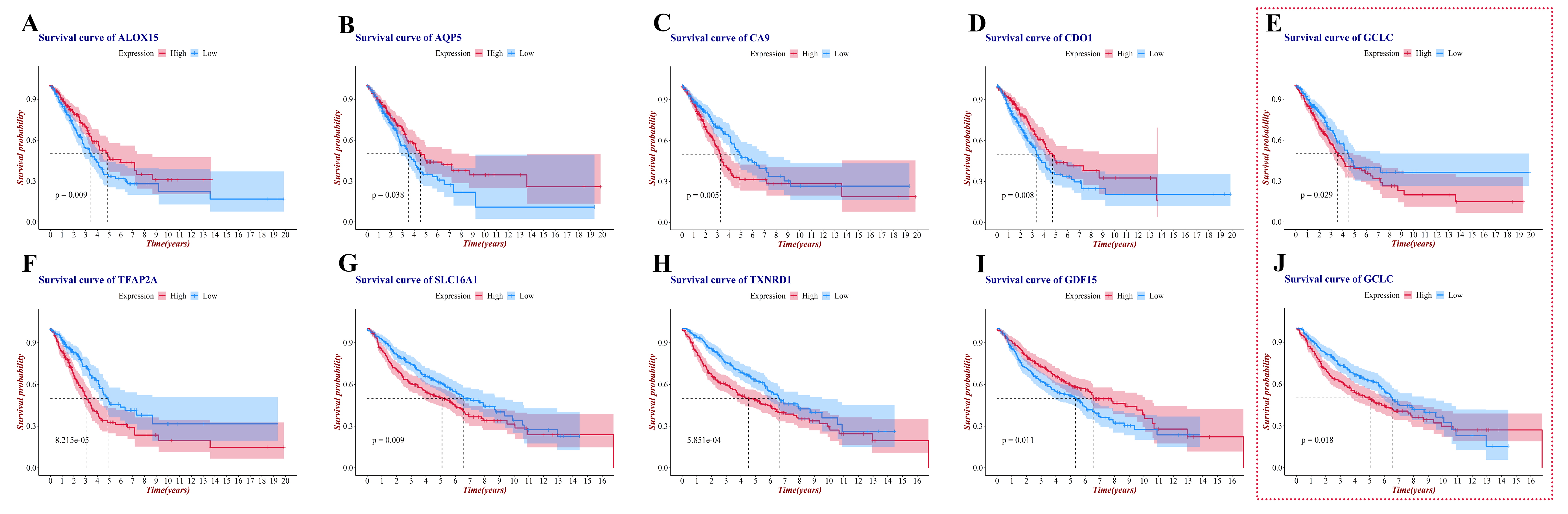

Supplement: Supplementary file 1 [file cells-11-03371-s001.zip › Supplementary figure2.tif]

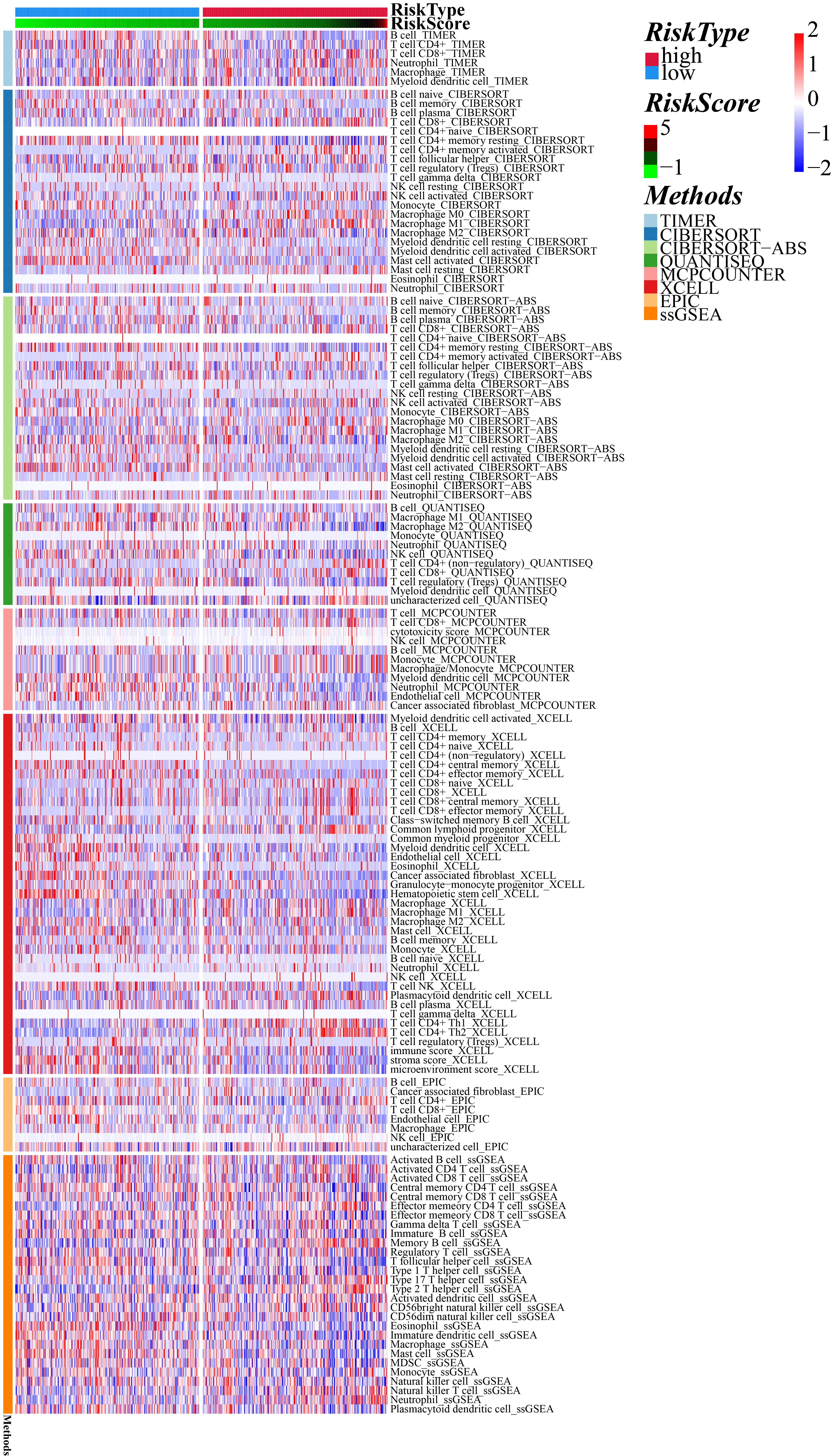

Supplement: Supplementary file 1 [file cells-11-03371-s001.zip › Supplementary figure3.tif]
